# Advanced Imaging of Shunt Valves in Cranial CT Scans with Photon-Counting Scanner

**DOI:** 10.3390/tomography10050050

**Published:** 2024-04-25

**Authors:** Anna Klempka, Eduardo Ackermann, Stefanie Brehmer, Sven Clausen, Christoph Groden

**Affiliations:** 1 Department of Neuroradiology, University Medical Centre Mannheim, Medical Faculty Mannheim, University of Heidelberg, 68167 Mannheim, Germany; 2 Department of Neurosurgery, University Medical Centre Mannheim, Medical Faculty Mannheim, University of Heidelberg, 68167 Mannheim, Germany; 3 Department of Radiation Oncology, University Medical Centre Mannheim, Medical Faculty Mannheim, University of Heidelberg, 68167 Mannheim, Germany

**Keywords:** photon counting, computer tomography, shunt valves

## Abstract

This brief report aimed to show the utility of photon-counting technology alongside standard cranial imaging protocols for visualizing shunt valves in a patient’s cranial computed tomography scan. Photon-counting CT scans with cranial protocols were retrospectively surveyed and four types of shunt valves were encountered: proGAV 2.0^®^, M.blue^®^, Codman Certas^®^, and proSA^®^. These scans were compared with those obtained from non-photon-counting scanners at different time points for the same patients. The analysis of these findings demonstrated the usefulness of photon-counting technology for the clear and precise visualization of shunt valves without any additional radiation or special reconstruction patterns. The enhanced utility of photon-counting is highlighted by providing superior spatial resolution compared to other CT detectors. This technology facilitates a more accurate characterization of shunt valves and may support the detection of subtle abnormalities and a precise assessment of shunt valves.

## 1. Introduction

Photon-counting computed tomography (PC CT) has already become a promising technique for the beneficial imaging of the heart and vessels, as well as small structures in the middle ear. Many reviews describe the technical potential of the improved iodine contrast-to-noise ratio or improved spatial resolution as promising for more applications. Spectral imaging grants even more space for future potential developments [1,2,3].

PC detectors represent a significant advancement in imaging technology, offering distinct differences and advantages over traditional so-called energy-integrating detectors (EIDs). The fundamental difference lies in their method of processing X-ray signals. While EIDs measure the total energy deposited by X-rays over a certain time period, providing an aggregated signal, PCDs count individual photon events, offering precise energy resolution for each detected photon. This key distinction enables PC detectors to directly quantify the number of X-rays absorbed and to discern between photons of different energies, allowing for material differentiation and multi-energy imaging. Such capabilities are particularly advantageous in medical imaging, enabling enhanced contrast, reduced dose, and improved diagnostic accuracy by distinguishing between different types of tissue and materials. In contrast, EIDs’ lack of energy discrimination leads to images that are based on overall attenuation without the ability to directly measure or exploit the energy information of the incoming photons. This often results in images that can suffer from contrast loss and increased noise, particularly at lower doses. Moreover, PC CT’s superior energy resolution reduces scatter and beam-hardening artifacts, which are common challenges in imaging with EIDs. The ability to operate at lower radiation doses without compromising image quality further underscores the difference, showcasing PC detectors as a transformative technology in the field of diagnostic imaging [4,5,6].

This research focused on shunt valves used to treat hydrocephalus. Hydrocephalus, caused by excessive cerebrospinal fluid accumulation in the brain’s ventricles, leads to increased intracranial pressure and brain damage. A common treatment is to surgically implant a shunt system to divert the cerebrospinal fluid elsewhere for absorption, such as the abdominal cavity. Modern shunt systems often have programmable valves which can be utilized to make unobtrusive adjustments to the rate of drainage. This enables customized treatment, improving patients’ outcomes and life quality.

Nowadays, there is an overwhelming number of valve manufacturers and in turn a myriad of valve design types available on the market. The lack of uniformity and standardisation presents a challenge in routine diagnostics [7]. The focus of our investigation was to analyse the data obtained from our institution and test the clinical potential of PC CT. At this stage, we refrained from comparing valves based on their manufacturing origin. The patient group in our study was diverse, representing various clinical scenarios, which is a typical feature of everyday practice. 

Some cerebrospinal fluid valves, such as M.blue and proGAV 2.0, are constructed with metallic material on both sides, and this presents an interesting challenge for imaging due to the need for high spatial resolution and the suppression of metal artifacts. Therefore, M.blue and proGAV 2.0 valves were included in our study to test the ability of PC CT imaging in terms of reducing artifacts, as well as its method specificity [8]. Previous studies have documented attempts at valve visualization through CT scans, predominantly focusing on the Certas Plus Valve. However, to our knowledge, no comprehensive studies have been conducted so far on the proGAV 2.0 or M.blue valves. Our research endeavours to fill this gap in the literature. In our detailed article, we report, for the first time, the successful imaging of both the M.blue and proGAV 2.0 valves within a clinical setting, employing meticulously defined scan parameters to ensure precision and clarity [9,10]. 

Our study diverged from conventional practices by opting for a standard CT scan of the neurocranium rather than resorting to navigational scans tailored for specific settings, such as adjusted angles towards the skull base or specialized thin-slice exposures. This approach allowed us to establish a baseline for standard imaging procedures in the presence of valves. Additionally, we have taken the initiative to quantify the radiation dose associated with these imaging techniques, an aspect that has been notably absent in prior discussions on this subject. Our findings not only contribute to the existing body of knowledge but also open new avenues for further research in the optimization of imaging practices for patients with shunt valves.

## 2. Materials and Methods

### 2.1. Data Collection

All patients had a clinical indication for a cranial CT scan based on the decision of the treating neurosurgeon. We retrospectively collected nine scans of ten shunt valves: five Codman Certas Plus Shunt systems from Integra LifeSciences (Princeton, NJ, USA), and the others from Miethke (Potsdam, Germany): three proGAV 2.0, one M.blue, and one proSA. There were nine patients: five females and four males (mean age 54.7 +/− 15.8). 

All scans were conducted using a cranial CT protocol on the PC CT scanner Naeotom Alpha Siemens Haelthineers (Forchheim, Germany) with the settings of 120 kV, quality reference 72 mAs, ME67, pitch factor 0.35, rotation time 0.5 s, and matrix size 512 × 512 using spiral acquisition. The radiation dose measured in terms of DLP (Dose-Length Product) was recorded for every patient. All scans were supplemented by energy-integrated CT scans from the same patient in a different time point. The scan protocols were consistent with clinically used head CT protocols on every tomograph. The scanners used were high-end energy-integrated scanners in our institution, with all but one scan utilizing spiral acquisition. The protocol characteristics were in line with clinically used head protocols. 

### 2.2. Imaging Reconstruction

The imaging reconstruction from each shunt valve was performed by an experienced radiologist, adapting the imaging to the specific characteristics of the shunt valve. Imaging of each valve was conducted in Syngo.via Client Software version 8.3, using the 3-dimensional multiplanar reconstructions mode, always with a hard kernel reconstruction of 1.0 mm slice thickness from all datasets and an increment equal or lower than 1.0 mm. In the 1 mm thick dataset with a hard kernel (for imaging cranial bones), in the first step, the valve was localized in coronal, sagittal, and axial images. Then, in the second step, the angulation was adjusted to match that of the valve. In the third step, imaging parameters such as the thickness of multiplanar reconstructions and the grayscale parameters, including window width and window centre, were adjusted to enhance the visibility of the markings for reading. 

### 2.3. Evaluation

**Figure 1:**
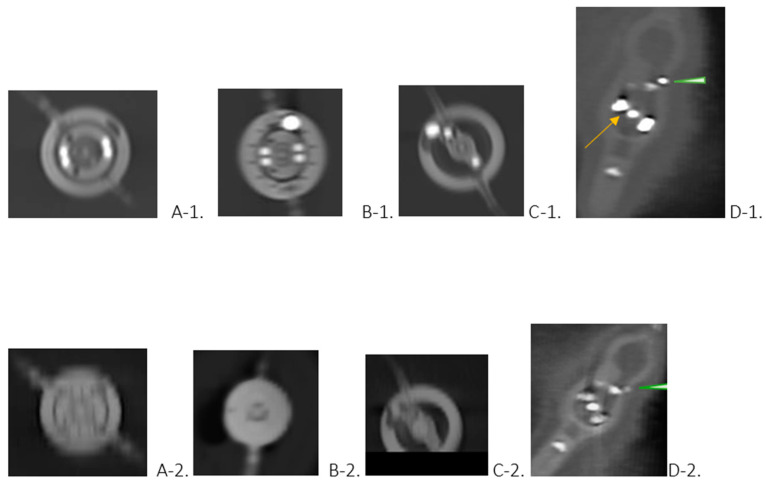
(**Upper row**)—Valves depicted with standard cranial protocol in photon-counting CT. (**Lower row**)—Multiplanar reconstructions obtained using CT scanners other than PC CT. The photos belong to 4 different patients (**A**–**D**). (**Upper row**)—Images of the proGav 2.0 (**A-1**), M. blue (**B-1**), proSA (**C-1**), and Codman Certas Plus (**D-1**) shunt valves, with each image orienting the upper edge as cranial and the lower edge as caudal. The clear visibility of the markings aids in determining the valve adjustments: (**A-1**) 13 mmH_2_O, (**B-1**) 5 mmH_2_O, (**C-1**) 14 mmH_2_O, and (**D-1**) Setting5 145 mmH_2_O ± 35 mmH_2_O. Each image was reconstructed from a dataset with a thickness of 1 mm with a hard kernel, resulting in an multiplanar reconstruction approximate thickness of 3 mm for images (**A-1**–**C-1**), and 1 mm for image (**D-1**). In image (**D-1**), particular attention is drawn to the magnet marking with a tantalum ball serving as a setting indicator (arrow) and a marker on the right-hand side (arrowhead). (**Lower row**)—ProGav 2.0 (**A-2**), M.blue (**B-2**), proSA (**C-2**), and Codman Certas Plus (**D-2**) shunt valves. However, the information associated with each image was only possible to discern after reviewing patients’ history due to the inability to properly identify the shunt valve and recognize adjustments solely based on the images. Only in image (**C-2**), depicting the proSA valve, was it possible to discern the markings and interpret the valve settings. Conversely, in image (**D-2**), featuring the Codman Certas Plus valve, the markings were not clearly visible, making it difficult to determine the location of the magnet marking. However, the right-hand side marker is visible (arrowhead).

We compared radiation doses and sought consensus between two experienced radiologists, based on each setting, to determine better imaging in terms of sharpness, contrast, and readiness of adjustments. Our observation is briefly presented in Figure 1, along with supplementary aspects from ex vivo imaging of three depicted shunt valves. 

## 3. Results

Our analysis, summarized in Figure 1, proved the high definition and accuracy of the PC CT portrayal of shunt valves. A uniformity of opinions among radiologists was observed in all cases, showing the higher confidence in interpretations with PC CT. The comparison of DLP from PC CT (675.3 ± 94.4) and from other scanners (716.78 ± 77.44) revealed that DLP was about 6.14% lower in those patients scanned with PC CT. 

**Figure 2:**
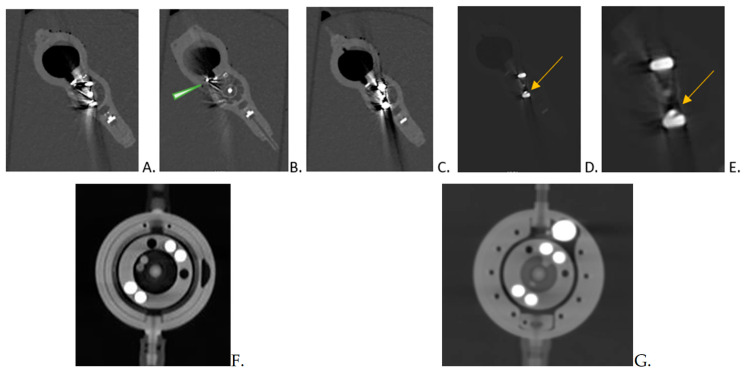
Ex vivo imaging to demonstrate the imaging possibilities of photon counting CT: Codman Certas (**A**–**E**) with proGAV (**F**) and M.blue (**G**) Codman Certas imPC CT imaging using slices of 0.5 mm thickness. A special emphasis is placed on the magnet marking, indicated by a tantalum ball serving as a setting indicator (arrow) in images (**C**,**E**), and a marker on the right-hand side (arrowhead) in image (**B**). It is important that the settings depicted in images (**D**,**E**) are consistent with those in the other pictures of the valve (**A**–**C**). Only using the unique capabilities of photon counting, which offer enhanced brightness and adjustments in grayscale, was it possible to accurately interpret the readings.

We added in vitro images of the three depicted shunts, along with ex vivo images that show excellent valve structures. These images were obtained using a clinically accepted protocol for imaging the temporal bone, and all three shunts were imaged with excellent capabilities. In Figure 2, we present additional images demonstrating the excellent ex vivo imaging of all structures of the valve.

## 4. Discussion

Identifying a patient’s valve is not a challenge when there is sufficient documentation; however, if the patient is not able to inform us about the shunt valve up to this point, skull X-rays are the standard method for identifying clinically unknown valve types and their settings reliably. The possibility of recognizing the shunt valve, in our observation, is feasible and not difficult with cranial PC CT. Moreover, the identification of other small implants and materials should be possible to conduct as well.

In all of the cranial scans of our patients obtained with the PC CT scanner, the visualisation of the shunt valves was precise. The three-dimensional imaging of the proGAV 2.0 and M.blue shunt valves was easier than visualizing the Codman valves, which demanded additional layers of detailing, as mentioned previously (Figure 2) [9,10]. This fact is firmly grounded in the fact that identifying valves has traditionally been the purview of X-ray imaging, and importantly, it involves displaying elements of varying densities within a single image.

It should be noted that all reconstructions were conducted using a hard kernel with 1 mm slice thickness, thus guaranteeing consistency and reliability in imaging processing. Some factors, such as brightness, have a significant impact on the quality of the images that were obtained. This can be seen, for example, in Figure 1(A-1), in contrast to A-2, where the levels of grayscale in CT of the head using an EID detector are different. In PC CT, additional aspects of the shunt valve, such as hyperdense markers, are much better visualized. A PC CT scan of the neurocranium enables the visualization of the intracranial situation in soft kernel reconstructions, shunt valve in hard kernel reconstructions, and shunt setting parameters in one single reconstruction. By overcoming the limitations associated with conventional CT imaging methods, PC offers superior visualization and diagnostic precision. This provides logistical advantages and saves time, stress, and radiation exposure for the patient. 

In the context of our preliminary research, we have enriched our findings by incorporating in vitro images of the three shunts. This addition aims to showcase the advanced capabilities of this imaging technique, as exemplified in Figure 2. Notably, the images reveal an exceptional level of detail, highlighting both the sharpness of the imaging process and a remarkable reduction in metal artifacts. These improvements are critical for enhancing diagnostic accuracy and potentially influencing treatment decisions, underscoring the value of our imaging approach in clinical practice.

Among the limitations of our brief study, however, are the facts that photon-counting CT is not available everywhere, and that, despite using lower doses of X-ray radiation compared to other scanners, it still has a higher radiation dose than traditional X-rays. There must also be specific indications for performing a CT scan other than the mere desire to assess valve settings with trained medical personnel.

This brief study highlighted the possibility for PC CT to overhaul shunt valve imaging in patients with hydrocephalus and potentially change the course of treatment. Contrary to the conventional imaging techniques which are limited in many ways, PC CT provides superior imaging and diagnostic accuracy. This advancement holds the potential of enhancing patient care by enabling more precise and timely diagnosis, as well as the management of hydrocephalus and its associated complications [11].

## 5. Conclusions

PC CT technology represents an exciting advancement in shunt valve imaging for hydrocephalus patients. By integrating this technology into standard cranial imaging protocols, clinicians may achieve superior visualization and characterization of shunt devices in a one-step modality with the necessary visualization of the intracranial structures. 

## Data Availability

The data presented in this study are available on reasonable request from the corresponding author.
